# Monoamine oxidase B inhibitor, selegiline, reduces ^18^F-THK5351 uptake in the human brain

**DOI:** 10.1186/s13195-017-0253-y

**Published:** 2017-03-31

**Authors:** Kok Pin Ng, Tharick A. Pascoal, Sulantha Mathotaarachchi, Joseph Therriault, Min Su Kang, Monica Shin, Marie-Christine Guiot, Qi Guo, Ryuichi Harada, Robert A. Comley, Gassan Massarweh, Jean-Paul Soucy, Nobuyuki Okamura, Serge Gauthier, Pedro Rosa-Neto

**Affiliations:** 1 Translational Neuroimaging Laboratory, The McGill University Research Centre for Studies in Aging, 6825 LaSalle Boulevard, Verdun, Québec H4H 1R3 Canada; 2 Department of Neurology, National Neuroscience Institute, 11 Jalan Tan Tock Seng, Singapore, 308433 Singapore; 3 Alzheimer’s Disease Research Unit, The McGill University Research Centre for Studies in Aging, McGill University, 6825 LaSalle Boulevard, Verdun, Québec H4H 1R3 Canada; 4 Montreal Neurological Institute/Hospital, Department of Pathology, McGill University Hospital Centre, 3801 University Street, Montreal, Québec H3A 2B4 Canada; 5 AbbVie Inc., 1 North Waukegan Road, North Chicago, IL USA; 6 Department of Pharmacology, Tohoku University Graduate School of Medicine, 2-1 Seiryo-machi, Aoba-ku, Sendai, 980-8575 Japan; 7 McConnell Brain Imaging Centre, McGill University, 3801 University Street, Montreal, Québec H3A 2B4 Canada; 8 Division of Pharmacology, Faculty of Medicine, Tohoku Medical and Pharmaceutical University, 4-4-1 Komatsushima, Aoba-ku, Sendai, 981-8558 Japan; 9 Montreal Neurological Institute, 3801 University Street, Montreal, Québec H3A 2B4 Canada; 10 Department of Neurology and Neurosurgery, McGill University, 3801 University Street, Montreal, Québec H3A 2B4 Canada

**Keywords:** ^18^F-THK5351 tau tracer, Monoamine oxidase-B, Selegiline, Alzheimer’s disease, Positron emission tomography

## Abstract

**Background:**

^18^F-THK5351 is a quinoline-derived tau imaging agent with high affinity to paired helical filaments (PHF). However, high levels of ^18^F-THK5351 retention in brain regions thought to contain negligible concentrations of PHF raise questions about the interpretation of the positron emission tomography (PET) signals, particularly given previously described interactions between quinolone derivatives and monoamine oxidase B (MAO-B). Here, we tested the effects of MAO-B inhibition on ^18^F-THK5351 brain uptake using PET and autoradiography.

**Methods:**

Eight participants (five mild cognitive impairment, two Alzheimer’s disease, and one progressive supranuclear palsy) had baseline ^18^F-AZD4694 and ^18^F-THK5351 scans in order to quantify brain amyloid and PHF load, respectively. A second ^18^F-THK5351 scan was conducted 1 week later, 1 h after a 10-mg oral dose of selegiline. Three out of eight patients also had a third ^18^F-THK5351 scan 9–28 days after the selegiline administration. The primary outcome measure was standardized uptake value (SUV), calculated using tissue radioactivity concentration from 50 to 70 min after ^18^F-THK5351 injection, normalizing for body weight and injected radioactivity. The SUV ratio (SUVR) was determined using the cerebellar cortex as the reference region. ^18^F-THK5351 competition autoradiography studies in postmortem tissue were conducted using 150 and 500 nM selegiline.

**Results:**

At baseline, ^18^F-THK5351 SUVs were highest in the basal ganglia (0.64 ± 0.11) and thalamus (0.62 ± 0.14). In the post-selegiline scans, the regional SUVs were reduced on average by 36.7% to 51.8%, with the greatest reduction noted in the thalamus (51.8%) and basal ganglia (51.4%). MAO-B inhibition also reduced ^18^F-THK5351 SUVs in the cerebellar cortex (41.6%). The SUVs remained reduced in the three patients imaged at 9–28 days. Tissue autoradiography confirmed the effects of MAO-B inhibition on ^18^F-THK5351 uptake.

**Conclusions:**

These results indicate that the interpretation of ^18^F-THK5351 PET images, with respect to tau, is confounded by the high MAO-B availability across the entire brain. In addition, the heterogeneous MAO-B availability across the cortex may limit the interpretation of ^18^F-THK5351 scans using reference region methods.

## Background

In vivo characterization of tau pathology using positron emission tomography (PET) constitutes a new frontier in Alzheimer’s disease (AD) research [1]. It is expected that tau imaging agents will provide the means for staging disease progression as well as selecting patients appropriate for a given therapy, confirming the mechanism of action of pharmacological interventions affecting tau aggregates and monitoring treatment efficacy for AD [2].

^18^F-THK5351 is a quinoline-derivative tau imaging tracer with affinity to paired helical filaments (PHF), a typical tau aggregate present in neurofibrillary tangles (NFTs). ^18^F-THK5351 retention in the temporal lobes in vivo is able to distinguish AD patients from healthy individuals [3]. However, ^18^F-THK5351 is also retained in the basal ganglia and other brain regions known to express negligible amounts of PHF, a phenomenon attributable to off-target binding, which raises concerns about the specificity of ^18^F-THK5351 for PHF. Rather than PHF, the basal ganglia typically express high concentrations of aminergic projections, neuroreceptors, and their degrading enzymes such as monoamine oxidase (MAO) [4, 5]. As such, it is plausible to hypothesize that MAO-B (EC 1.4.3.4) constitutes a ^18^F-THK5351 off-target binding site, particularly given its previous reported affinity to quinolone derivatives [6]. Furthermore, the presence of high concentrations of MAO-B in the human cortex demands a careful examination of the impact of MAO-B binding on ^18^F-THK5351 images.

Selegiline is an irreversible MAO-B inhibitor utilized to enhance dopaminergic neurotransmission in Parkinson’s disease patients [7]. Previous PET studies revealed a persistent MAO-B inhibition following a single administration of MAO-B inhibitors [4]. As such, an acute selegiline challenge constitutes an interesting strategy to probe the contribution of brain MAO-B availability to the ^18^F-THK5351 uptake observed in patient populations.

Here, in a longitudinal observation of patients with baseline and post-selegiline ^18^F-THK5351 PET scans, we tested the hypothesis that a reduction of MAO-B availability also reduces ^18^F-THK5351 uptake. We further tested this hypothesis in postmortem human tissue using autoradiography.

## Methods

### Subjects

Eight individuals (five mild cognitive impairment (MCI), two AD, and one progressive supranuclear palsy (PSP)) were recruited. All study participants were screened to exclude any underlying neuropsychiatric diseases such as depression and concomitant intake of MAO inhibitors. Study participants then underwent a Mini-Mental State Examination (MMSE) [8] and Montreal Cognitive Assessment (MoCA) [9]. The MCI, AD, and PSP diagnoses were made using the Petersen’s [10], National Institute of Neurological and Communicative Disorders and Stroke and the Alzheimer’s disease and Related Disorders Association (NINCDS-ADRDA) [11], and the National Institute of Neurological Disorders and Stroke and the Society for PSP (NINDS-SPSP) [12] criteria, respectively.

### Scanning protocol

All eight participants had baseline ^18^F-AZD4694 and ^18^F-THK5351 PET scans to quantify brain amyloid and PHF load, respectively. A second ^18^F-THK5351 scan was conducted 1 week later, 1 h after an oral dose of 10 mg selegiline. Participants were also invited to undergo an optional third ^18^F-THK5351 PET scan 10 days after the selegiline administration. Each ^18^F-THK5351 acquisition consisted of dynamic images (4 × 5 min) acquired at 50–70 min after intravenous bolus injection. The mean ± standard deviation injected radioactivity of ^18^F-THK5351 was 6.6 ± 0.3 mCi for the baseline scan, 6.7 ± 0.4 mCi for the post-selegiline scan, and 6.9 ± 0.4 mCi for the third scan. ^18^F-AZD4694 acquisition consisted of dynamic images (6 × 5 min) acquired at 40–70 min after intravenous bolus injection of ^18^F-AZD4694. The injected radioactivity of ^18^F-AZD4694 was 6.3 ± 0.3 mCi. A 6-min transmission scan was acquired at the end of each PET scan. All PET scans were performed using the Siemens High Resolution Research Tomograph (HRRT). A magnetic resonance imaging (MRI) anatomical scan was also performed for all patients using the 1.5 T Siemens Sonata scanner for co-registration purposes.

### ^18^F-THK5351 and ^18^F-AZD4694 synthesis

^18^F-THK5351 was synthesized according to a previously published paper [3], with an average specific activity of 24.3 Ci/μmol for the baseline scan, 19.2 Ci/μmol for the post-selegiline scan, and 13.5 Ci/μmol for the third scan. The average injected mass dose was 5.58 ng/kg for the baseline scan, compared with 6.68 ng/kg for the post-selegiline scan (*P* = 0.61, not significant), and 4.21 ng/kg for the third scan (*P* = 0.39, not significant). ^18^F-AZD4694 was synthesized using a modified synthesis procedure previously described [13], with an average specific activity of 6.1 Ci/μmol.

### PET and MRI image processing

T1-weighted MRI images were processed using the CIVET image processing pipeline [14, 15] for field distortions correction, and the PET images were processed using an established image processing pipeline [16]. Briefly, using transformations obtained from PET native to MRI native space and the MRI native to the Montreal Neurological Institute (MNI) 152 space, the PET images were linearly registered and subsequently spatially normalized to the MNI 152 standardized space. The primary outcome measure was the standardized uptake value (SUV). SUVs were calculated using tissue radioactivity concentration data from 50 to 70 min following ^18^F-THK5351 injection, and 40 to 70 min following ^18^F-AZD4694 injection, normalized by body weight and injected radioactivity. The baseline and post-selegiline ^18^F-THK5351 SUVs in the cortical gray matter of the frontal, parietal, lateral temporal, and occipital lobes, hippocampus, cingulate gyri, basal ganglia, thalamus, and cerebellar cortex of each individual were measured. We then calculated the average percentage reduction of mean regional SUV in the post-selegiline scans. The ^18^F-THK5351 SUV ratio (SUVR) and ^18^F-AZD4694 SUVR were obtained using the cerebellar cortex and cerebellum as reference regions, respectively. The ^18^F-AZD4694 SUVR images were assessed by two independent experts (JPS and PRN) to visually classify the participants as amyloid-positive or amyloid-negative after full agreement was reached. In a clinical validation of ^18^F-AZD4694 using the cerebellum as the reference region, the SUVRs in AD patients varied between 2.10 and 2.88 across regions [13].

### Autoradiography

In vitro autoradiography was performed on 14 postmortem brain sections (seven AD patients with a diagnosis confirmed by pathological assessment, and seven age-matched healthy controls) as previously described [17]. In brief, baseline and R-(–)-deprenyl hydrochloride competition experiments were performed on adjacent brain sections (20 μm thickness). Prepared frozen brain tissues were warmed to room temperature, air-dried, and pre-incubated in a buffer saline solution (138 mM NaCl and 27 mM KCl, pH 7.4 adjusted by NaOH) and 1% bovine serum albumin for 20 min. Brain tissues were then air-dried and incubated with ^18^F-THK5351 (1.85 μCi) and the R-(–)-deprenyl hydrochloride challenge was performed at concentrations of 150 nM and 500 nM for 150 min. After the incubation, brain tissues were dipped three times in the buffer solution for 3 min each time, once in 4 °C water for 30 s, and dried under a stream of cool air. The brain sections were subsequently exposed to a radioluminographic imaging plate (Fujifilm BAS-MS2025) for 20 min and obtained using FUJIFILM BAS-5000. Activity in photostimulated luminescence units per mm^2^ was measured using Image Gauge 4.0 (Fujifilm) and the percentage of ^18^F-THK5351 total binding after R-(–)-deprenyl hydrochloride challenge was calculated.

### Statistical analysis

The statistical analysis was performed using GraphPad Prism Version 7.0b (GraphPad Software, La Jolla, California, USA; www.graphpad.com). The baseline and the post-selegiline regional ^18^F-THK5351 SUVs were compared using paired *t* test analyses. The Bonferroni correction was used to correct the aforementioned analyses for multiple comparisons.

## Results

**Fig. 1:**
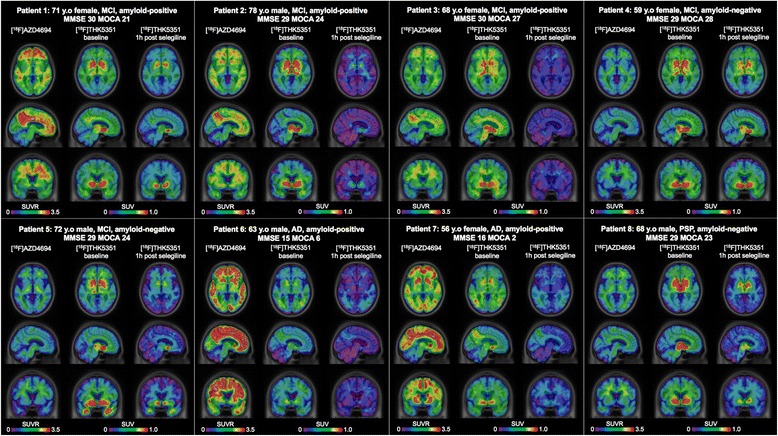
Selegiline reduces brain ^18^F-THK5351 standardized uptake value. Standardized uptake value (*SUV*) and SUV ratio (*SUVR*) map overlaid on a structural MRI scan showing the SUVR from 40 to 70 min after ^18^F-AZD4694 injection, and baseline and 1 h post-selegiline SUV from 50 to 70 min after ^18^F-THK5351 injection in the eight patients. *AD* Alzheimer’s disease, *MCI* mild cognitive impairment, *MMSE* Mini-Mental State Examination, *MoCA* Montreal Cognitive Assessment, *PSP* progressive supranuclear palsy, *y.o* years old

Baseline demographics and global ^18^F-AZD4694 SUVR of the study participants are summarized in Table 1. Patients 4, 5 and 8 are classified as amyloid-negative and patients 1, 2, 3, 6, and 7 are classified as amyloid-positive (Fig. 1).

**Table 1:** Baseline demographics

	Patient 1	Patient 2	Patient 3	Patient 4	Patient 5	Patient 6	Patient 7	Patient 8
Diagnosis	MCI	MCI	MCI	MCI	MCI	AD	AD	PSP
Age (years)	71	78	68	59	72	63	55	68
Gender	Female	Male	Female	Female	Male	Male	Female	Male
MMSE	30	29	30	29	29	15	16	29
MoCA	21	24	27	28	24	6	2	23
Global ^18^F-AZD4694 SUVR	2.19	1.89	1.73	0.96	0.94	2.64	2.24	1.04

*AD* Alzheimer’s disease, *MCI* mild cognitive impairment, *MMSE* Mini-Mental State Examination, *MoCA* Montreal Cognitive Assessment, *PSP* progressive supranuclear palsy, *SUVR* standardized uptake value ratio

**Fig. 2:**
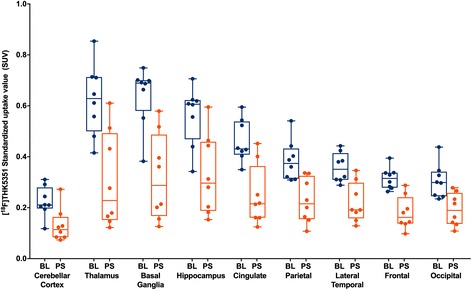
Regional ^18^F-THK5351 standardized uptake value declines in the post-selegiline scans. Whisker plot showing significant differences between baseline and 1 h post-selegiline regional standardized uptake value (*SUV*) in the brain regions 50 to 70 min after ^18^F-THK5351 injection in the eight patients. Cerebellar cortex: *P* = 0.001; thalamus: *P* = 0.001; basal ganglia: *P* < 0.001; hippocampus: *P* = 0.003; cingulate: *P* < 0.001; parietal: *P* < 0.001; lateral temporal: *P* = 0.001; frontal: *P* < 0.001; occipital: *P* < 0.001. *BL* baseline, *PS* post-selegiline

The ^18^F-THK5351 SUV map demonstrated an average of 36.7 to 51.8% regional uptake reduction in the post-selegiline scans from the baseline scans (Fig. 1). At baseline, the mean SUVs were highest in the basal ganglia (0.64 ± 0.11) followed by the thalamus (0.62 ± 0.14) (Fig. 2). In the post-selegiline scans, there were statistically significant regional SUV declines compared to the baseline scans. The SUV reduction was greatest in the thalamus (51.8%), followed by the basal ganglia (51.4%). MAO-B inhibition also reduced ^18^F-THK5351 SUVs in the cerebellar cortex (41.6%).

**Fig. 3:**
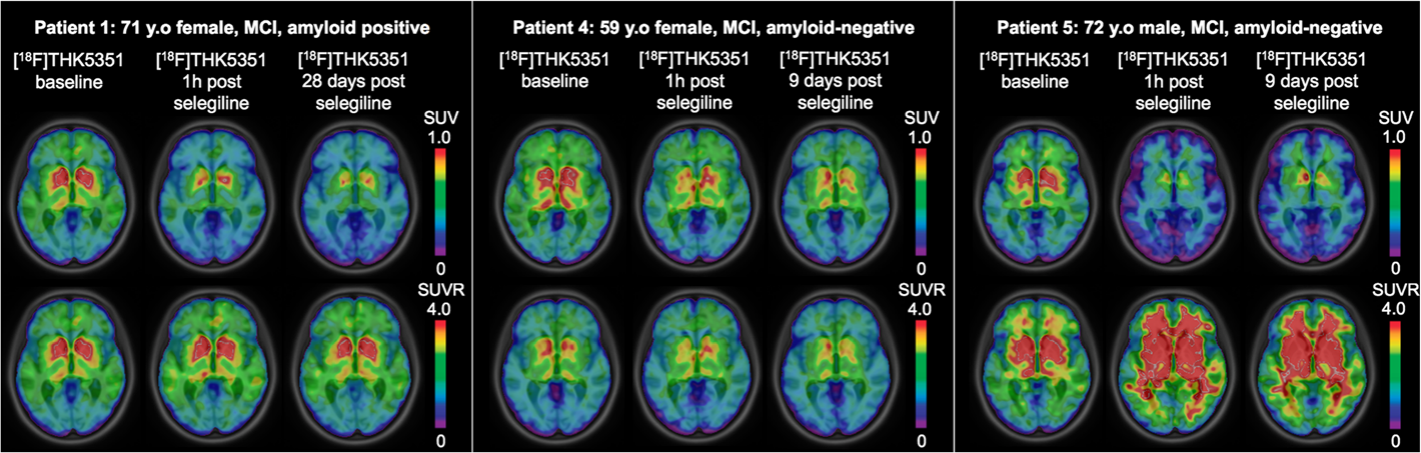
The reduction of ^18^F-THK5351 standardized uptake value remains in the second follow-up. Standardized uptake value (*SUV*) and SUV ratio (*SUVR*) map overlaid on a structural MRI scan showing the baseline, 1 h and 9–28 days post-selegiline SUV and SUVR from 50 to 70 min after ^18^F-THK5351 injection in three patients. *MCI* mild cognitive impairment, *y.o* years old

**Fig. 4:**
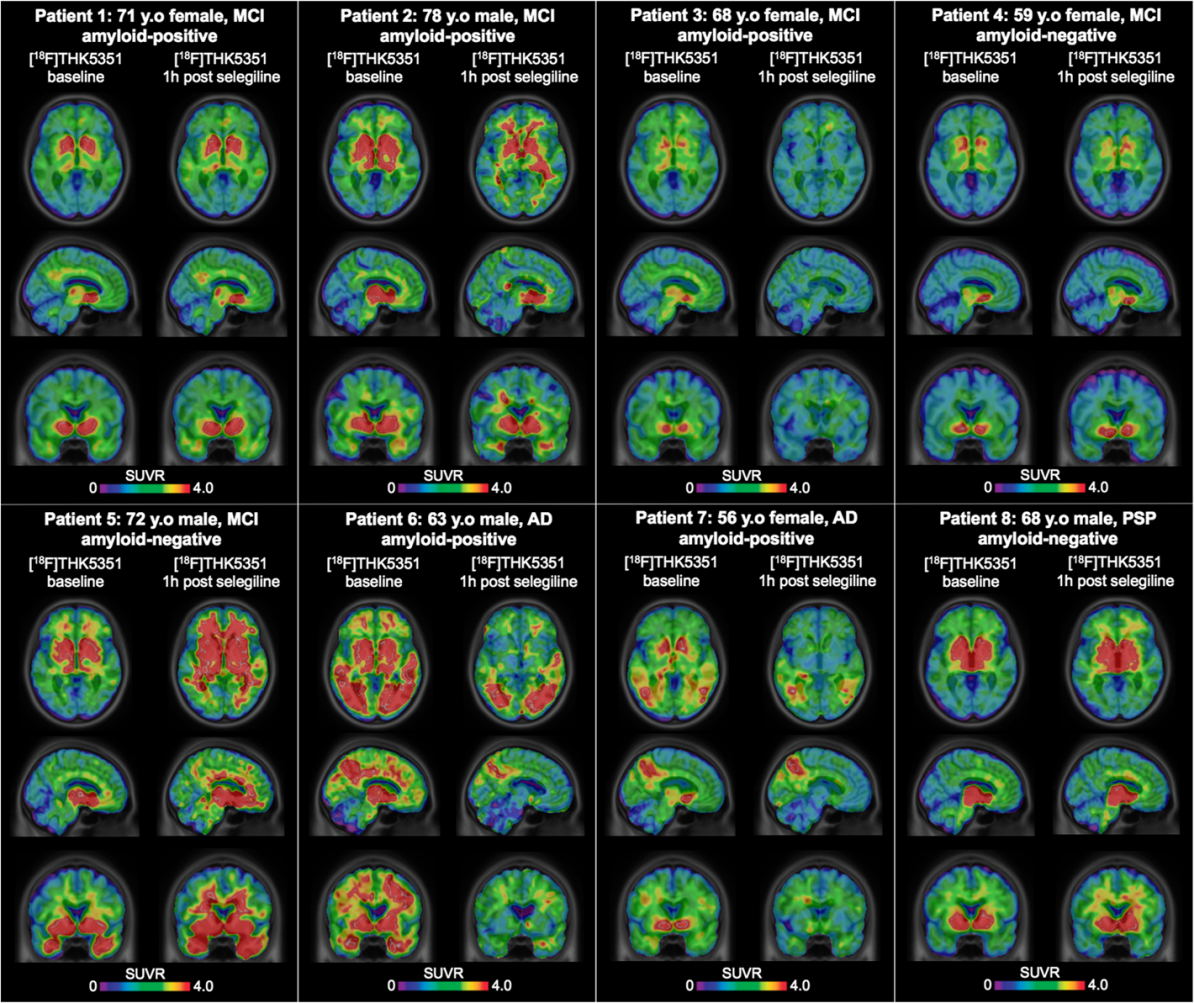
The effects of selegiline in cerebellum mask the reduction of ^18^F-THK5351 standardized uptake value ratio. Standardized uptake value ratio (*SUVR*) map overlaid on a structural MRI scan showing the baseline and 1 h post-selegiline SUVR from 50 to 70 min after ^18^F-THK5351 injection in the eight patients. *AD* Alzheimer’s disease, *MCI* mild cognitive impairment, *PSP* progressive supranuclear palsy, *y.o* years old

In the three MCI individuals who underwent a third ^18^F-THK5351 PET scan 9–28 days after the selegiline administration, the SUV remained reduced (Fig. 3). There was no consistent regional ^18^F-THK5351 SUVR reduction in the post-selegiline scans from baseline (Figs. 3 and 4), which is in line with the anticipated differences in the PHF to MAO-B ratio in different brain regions of different participants, including the cerebellar cortex reference region.

**Fig. 5:**
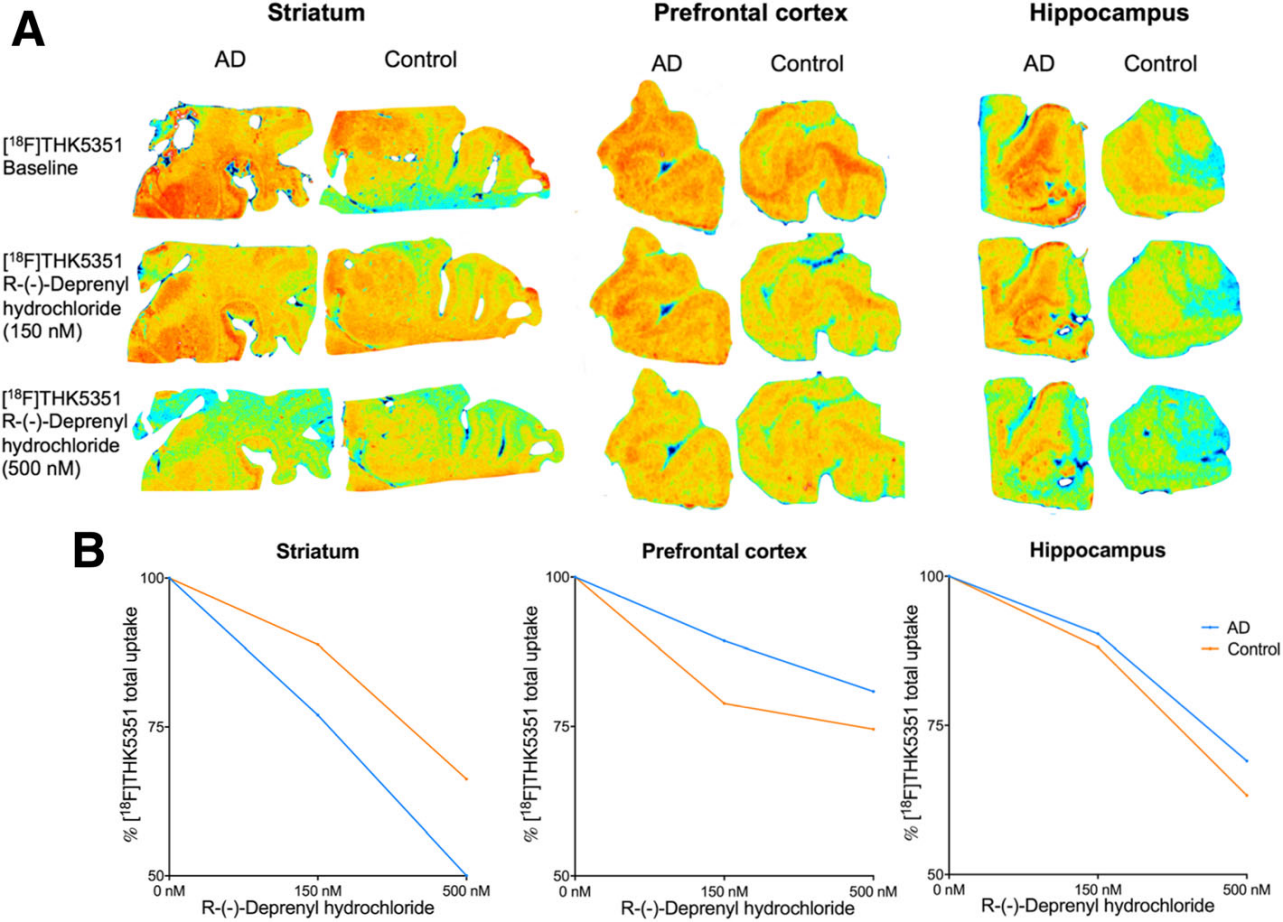
^18^F-THK5351 autoradiography competition with R-(–)-deprenyl hydrochloride shows dose-dependent uptake decline. **a** In vitro autoradiography of the postmortem striatum, prefrontal cortex, and hippocampal brain sections of Alzheimer’s disease (*AD*) patients and controls showing the reduction of baseline ^18^F-THK5351 binding after 150 nM and 500 nM R-(–)-deprenyl hydrochloride challenge. **b** Reduction of percentage of ^18^F-THK5351 total uptake is greater in the striatum than in the prefrontal cortex and hippocampus

Autoradiography in postmortem brain sections of AD patients and healthy controls further demonstrated a reduction of total ^18^F-THK5351 uptake following 150 nM and 500 nM R-(–)-deprenyl hydrochloride challenge (Fig. 5a). This reduction was greater in the striatum, a region rich in MAO-B but with negligible PHF, than in the prefrontal cortex and hippocampus where both PHF and MAO-B are present (Fig. 5b).

## Discussion

In summary, we showed that brain and cerebellar cortex MAO-B availability affects ^18^F-THK5351 SUV. Following a single 10 mg oral dose of selegiline, ^18^F-THK5351 SUVs decreased by approximately 30 to 50% depending on the brain region, with the highest decline noted in the basal ganglia and thalamus which are known to express the highest concentrations of MAO-B in the brain [4]. The in vitro autoradiography blocking experiments showed similar effects. The long-lasting selegiline-evoked ^18^F-THK5351 SUV decline indicates MAO-B inhibition as the possible mechanism underlying this effect.

MAO-B is a protein highly expressed in all brain regions. It is compartmentalized in the outer membrane of the astrocytes mitochondria [18]. During the normal aging process, global MAO-B availability increases at the rate of nearly 9% per decade [19, 20]. In fact, MAO-B imaging has been proposed as a biomarker for astrocytosis in various neurodegenerative conditions associated with cell death or activation of immune responses, with some investigators reporting higher MAO-B binding in AD patients versus aged-matched controls [21–23]. Moreover, MAO-B inhibition has significant therapeutic effects for mood disorders and Parkinson’s disease, and it is considered a therapeutic target for various neurodegenerative conditions [24].

The high retention of tau imaging agents such as ^18^F-THK5351 and ^18^F-flortaucipir to brain structures known to be devoid of PHF has been attributed to off-target binding sites. It is important to emphasize that off-target binding implies specific (saturable) binding of the imaging agent to proteins other than the primary target for which it was intended. Generally, it is desirable that the affinity of the target binding sites be higher by at least one order of magnitude as compared to that of the off-target binding sites, although the lack of such a specificity might be acceptable if the target and off-target binding sites do not coexist in the same brain region [25]. Our results suggest that ^18^F-THK5351 binds to MAO-B and PHF with similar affinity, which might be detrimental to PHF quantification in the brain. However, given that ^18^F-THK5351 is biochemically different from ^18^F-flortaucipir, our findings cannot be generalized.

Interestingly, the acute selegiline challenge conducted in this study evoked the highest ^18^F-THK5351 SUV decline in the thalamus and striatum, which express high levels of MAO-B [4], and are also known to be devoid of PHF. However, it is worth mentioning that the magnitude of MAO-B inhibition in the aforementioned regions was variable, as described by previous studies [26, 27]. This variability can be attributed to individual differences in selegiline absorption, biodistribution, or pharmacodynamics. Furthermore, the exposure to MAO-B inhibitors present in tobacco, certain antidepressants, or herbal therapies may also influence such variability [28, 29].

The long-lasting effects of selegiline support MAO-B inhibition as the main mechanism involved in the effects observed by this study. Indeed, previous studies conducted in vivo estimate that the de novo synthesis half-life of MAO-B is 40 days [30]. Our autoradiography results conducted in postmortem brain of AD patients (Fig. 5) also support ^18^F-THK5351 binding to both MAO-B and PHF. Importantly, the enrolment of a clinically heterogeneous population in this study was intended to test whether MAO-B availability affects ^18^F-THK5351 SUV independently of other specific pathophysiological processes.

The selegiline challenge in the present study affected cortical ^18^F-THK5351 SUVs (Fig. 1) in regions known to accumulate PHF, such as the mesial basal temporal, temporal, and parietal cortices [31]. These regions also express high concentrations of MAO-B [5]. The visual assessments of ^18^F-THK5351 SUV images obtained in amyloid-positive individuals revealed the signature previously described in AD patients [2], which remained after the challenge with selegiline. Similarly, the high uptake in the midbrain and basal ganglia of our patient with PSP remained after the post-selegiline scan. This may support the concept that ^18^F-THK5351 binds to 4-R tau pathology in the midbrain and basal ganglia, which is consistent with the imaging signature recently described [32]. The AD and PSP signatures observed after the selegiline challenge represent images with decreased effects of MAO-B availability.

We found that cerebellar cortex ^18^F-THK5351 SUVs declined to a lesser extent as compared to cerebral cortical regions following the selegiline challenge. This observation is consistent with previous studies showing declines in cerebellar MAO-B availability following pharmacological inhibition [5, 33]. In our study, the quantification of ^18^F-THK5351 uptake using SUVR with the cerebellar cortex as a reference region was also sensitive to the selegiline challenge. Although cerebellar MAO-B availability invalidates the use of this region as a reference to derive ^18^F-THK5351 SUVR in the context of this study, future research should focus on methodology to correct MAO-B bias in ^18^F-THK5351 SUVR measurements [34].

The use of imaging agents with low specificity is acceptable to analyze PET images from brain regions without off-target binding sites [35, 36]. However, the interpretation of PET data obtained using tracers with low specificity becomes challenging when target and off-target binding sites co-localize within specific anatomical regions. In this situation, the major issue is obtaining PET signals of interest without the confounding effects from off-target sites. Tracer specificity becomes even more important in the case of imaging agents targeting abnormal protein aggregates. At early stages of the disease, when the load of abnormal protein aggregates is negligible, as compared to the availability of off-target binding sites, one could expect a higher confounding signal from the off-target binding [5, 37]. By contrast, in later stages of the disease, the relative contribution of off-target binding is reduced given the higher concentrations of protein aggregates in the tissue, despite high concentrations of MAO-B in the hippocampus, temporal neocortex, and cingulate gyrus [5]. It is also worth noting that the visual appearance of ^18^F-THK5351 PET images is not inconsistent with the pathological distribution of NFTs reported in Braak staging [38]. The potential reason for this is that, as neurons are compromised by NFTs, astrocytosis ensues in these regions [31]. The increase of MAO-B is likely due to astrocytosis in response to NFTs, and the PET signals here could be considered as a combination of both PHF and astrocytosis. This would need to be verified by future studies.

The interpretation of these results should take into consideration the following methodological limitations. Firstly, the present study was not conducted using full dynamic scans with an arterial input function, but relied on the SUV measured on ^18^F-THK5351 images obtained from 50 to 70 min after tracer injection [3] which is susceptible to physiological sources of variability. Secondly, although previous studies show that chronic administration of selegiline does not significantly affect cerebral blood flow, it has been reported that acute selegiline administration may induce rapid brain vasodilation via nitric oxide release [39]. Hence, it is possible that the selegiline dose in the present study may induce cerebral blood flow changes and affect accurate quantification of ^18^F-THK5351 uptake.

## Conclusions

The present study supports the concept that MAO-B is an ^18^F-THK5351 off-target binding site. As such, the current methods for analyzing ^18^F-THK5351 inevitably incorporate undesired confounding signal from MAO-B availability, which probably dominates PET signals in early stages of AD. Therefore, it would be appropriate for the methods used to quantify ^18^F-THK5351 to take into account the cortical MAO-B signal. Importantly, heterogeneous MAO-B availability across the cortex may also limit the quantification of ^18^F-THK5351 uptake using reference brain regions. In addition, with regards to the use of ^18^F-THK5351 in longitudinal studies and clinical trials in AD, one should be aware that both MAO-B and tau binding may increase with age at different rates in different brain regions of different individuals, whilst tau binding will increase in the same fashion with disease progression, or potentially decrease in response to an efficacious treatment. As such, experiments should be designed and interpreted with care to account for these processes. Finally, it is important to emphasize that the confounding effect of MAO-B availability in patients with high loads of PHF should be further evaluated by subsequent studies.

## Abbreviations

AD: Alzheimer’s disease
HRRT: High-Resolution Research Tomograph
MAO: Monoamine oxidase
MAO-B: Monoamine oxidase B
MCI: Mild cognitive impairment
MMSE: Mini-Mental State Examination
MNI: Montreal Neurological Institute
MoCA: Montreal Cognitive Assessment
MRI: Magnetic resonance imaging
NFT: Neurofibrillary tangle
NINCDS-ADRDA: National Institute of Neurological and Communicative Disorders and Stroke and the Alzheimer’s disease and Related Disorders Association
NINDS-SPSP: National Institute of Neurological Disorders and Stroke and the Society for PSP
PET: Positron emission tomography
PHF: Paired helical filaments
PSP: Progressive supranuclear palsy
SUV: Standardized uptake value
SUVR: Standardized uptake value ratio
